# Reply to Abrantes et al. Recombination-Based Perspectives on Lagovirus Classification, Phylogenetic Patterns, and Evolutionary Dynamics. Comment on “Shah et al. Genetic Characteristics and Phylogeographic Dynamics of Lagoviruses, 1988–2021. *Viruses* 2023, *15*, 815”

**DOI:** 10.3390/v16060928

**Published:** 2024-06-07

**Authors:** Pir Tariq Shah, Li Xing

**Affiliations:** 1 Faculty of Medicine, School of Biomedical Engineering, Dalian University of Technology, No. 2 Linggong Road, Dalian 116024, China; 2 Shandong Laboratory of Yantai Drug Discovery, Bohai Rim Advanced Research Institute for Drug Discovery, Yantai 264000, China; 3 Institute of Biomedical Sciences, Shanxi University, 92 Wucheng Road, Taiyuan 030006, China; 4 Shanxi Provincial Key Laboratory of Medical Molecular Cell Biology, Shanxi University, 92 Wucheng Road, Taiyuan 030006, China

Recently, Abrantes et al. commented on our published article “Genetic Characteristics and Phylogeographic Dynamics of Lagoviruses, 1988–2021”, Viruses, 15(4), 815 [1]. They raised several questions related to our results and conclusions, which are also important issues in the general field of virus evolution and worthy of being addressed to achieve further clarification. However, we believe that the authors have misunderstood many aspects of our article, especially the evolution of viruses. We presented a new perspective on the clustering, classification, phylogeographic dynamics, and evolution of lagoviruses based on the full-length genomes of the virus [1]. Performing new analyses to update the existing classification is quite common in the virus research field. Therefore, researchers in each article present new perspectives based on different analyses and use different genetic data to approach a more reliable and simpler conclusion as much as possible. This is the case with lagoviruses as well, where there is no classification system on the clade and sub-clade levels according to the ICTV (International Committee on Taxonomy of Viruses), and the phylogenetic trees were widely deduced using the partial genomic sequences of VP60 [2]. The GI.1/GI.2 taxonomy is the most recent, according to the structural protein nucleotide sequence-based phylogenetic relationships, where GI.1 was further divided into GI.1a (proposed as G6/RHDVa), GI.1b (G1), GI.1c (G2), and GI.1d (G3–G5) [3,4]. Similarly, several other classification and nomenclature systems were also cited by Abrantes et al., which together make lagoviruses’ classification complicated. Astonishingly, Abrantes et al. claimed that our classification makes the classification system complicated, which we disagree with. In fact, they have made it more complicated by comparing all the different classifications in the hope of achieving a consensus without considering the rapid evolution of viruses and advancement in the new tools and techniques for understanding the virus evolution. We utilized the latest, easily accessible, and reliable tools to make the classification easier and more reliable.

We provided the latest phylogeny based on full-length (complete) genome sequences using the latest tool (IQ tree) with 1000 bootstraps and the best-fitting models [5]. Furthermore, advancements in genetic sequencing and analysis tools have made sequencing the whole viral genome relatively inexpensive and quick, which helps in investigating the phylogenetic relationships between the different viruses within the family and between the virus strains arising in different hosts and geographic regions worldwide. Therefore, a full-length genome-based phylogeny is more reliable than partial genome-based phylogenies, while partial sequences are helpful for quickly identifying and verifying the virus. Further, Abrantes et al. think that the near-complete genome sequences without the 5′ and 3′ end primer binding regions should have been included in our analysis. As we know, the near-complete sequence is not a complete genome sequence and does not meet the criteria requirement for our analysis of full-length sequences. The inclusion of near-complete genome sequences might introduce a higher degree of variability due to potential gaps or uncertainties in the regions that are not covered. Thus, we included all the full-length genomes besides the VP60 coding sequences for comparison.

Abrantes et al. cited a published article stating that recombination causes the tree topology to change; thus, recombinants should not be included when classifying viruses. However, this idea is not correct. Genetic recombination is crucial in the evolution of viruses and plays a significant role in understanding and generating the diversity of viruses. In microorganisms, especially viruses, recombination is widespread [6]. In fact, non-recombinant microorganisms are quite rare [7], and recombination can also vary significantly between species [6,8,9,10]. Thus, we must know the position of the recombinants in the phylogenetic tree to track their evolution, pathogenicity, and immune escape, etc. Recombination is a natural force and plays a role in almost all viruses. The fact that recombination causes the tree topology to change does not disqualify the recombinant from appearing in the phylogenetic analysis. In our recombination analysis [1], we considered and presented only six recombinants, each with their major and minor parents. However, these are only the representatives of six recombination events, as we mentioned [1], which represent six different modes of recombination instead of only six recombinant viruses in the whole dataset. The major or minor parent virus listed in each recombination event is also, by default, a representative of a number of potential major or minor parents for each recombinant. In addition, the major or minor parent may also be the actual recombinant, making almost every strain a potential recombinant. Failing to understand the recombination detection analysis also led Abrantes et al. to conclude that we missed several previously reported recombinant strains in our study (please refer to the RDP4 software package guidelines for a better understanding [11]). Therefore, we cannot exclude and ignore the recombinants during the study of virus classification, characterization, and evolution.

In addition, Abrantes et al. have failed to understand the scale bar of the 30.0 nucleotide substitutions/site shown in the article [1]. We mentioned that the scale bar represents the nucleotide substitutions/site of the unrooted tree set to proportional. Also, they have identified that the FRG-USA strain (GenBank ID NC_001543.1) and FRG-Germany strain (GenBank ID M67473.1) are the same and not two different strains. To clarify these issues, we re-inferred the full-length ML (maximum likelihood) phylogenetic tree of all the strains (n = 239; FRG-USA strain excluded) using the IQ-TREE multicore version 1.6.12 with 1000 bootstraps and the best-fitting substitution model SYM+I+G4, while the tree branches were tested with the Shimodaira–Hasegawa-like approximate likelihood ratio test (SH-like aLRT) with 1000 replicates [5,12]. Our resulting unrooted trees are consistent with our already published one, dividing all the strains into four major clades, e.g., GI.1 (classical RHDV), GI.2 (RHDV2), HaCV/EBHSV, and RCV (Figure 1A–C; Supplementary Figure S1). Similarly, the midpoint-rooted tree exhibited four separate clades: GI.1 (classical RHDV), GI.2 (RHDV2), HaCV/EBHSV, and RCV. However, two of the RHDV strains, e.g., RHDV_GER-NW_EI17-1.L03577 and RHDV_GER_NW_D144-2.L01046 (GenBank IDs LR899142.1 and LR899187.1, respectively), appeared as separate clades (Figure 1D) rather than together with HaCV/EBHSV (Figure 1A–C; Supplementary Figure S1) [1]. In addition, CBMad17-1 (GenBank ID MF407655.1, Portugal-2017-Rabbit) appeared as a distinct strain closer to the RCV strains within all four types of phylogenetic representations. Although the tree is represented with different visualization methods, the overall topology remains the same, e.g., dividing all the strains into four major clades as follows: GI.1 (classical RHDV), GI.2 (RHDV2), HaCV/EBHSV, and RCV. However, the scale bar of the nucleotide substitutions/site is 30.0 for the unrooted proportional tree (Figure 1A), 0.2 for the unrooted unchanged tree (Figure 1B), 3.0 for the unrooted circular cladogram tree (Figure 1C), and 0.1 for the midpoint-rooted tree (Figure 1D). These are different types of representations of a phylogenetic tree, expressing different nucleotide substitutions/site values, where the methods exhibit the same conclusion. Further understanding of the molecular phylogenetics can be gained by referring to [13,14,15]. To avoid making the classification more complex, we chose the simple way of presenting the lagovirus clades and sub-clades to provide an easy-to-understand picture of the lagovirus classification on the clade and sub-clade levels.

Moreover, Abrantes et al. mentioned that we missed citing some nicely demonstrated articles. However, nice is not the standard for citing a scientific work. The articles unrelated to our understudy point do not necessarily need to be cited. Also, they claimed that we have not cited countless papers. We cannot cite all the papers in a research article as it will divert the discussion from the main findings and results of the research paper. The purpose of a research article is to present updates to and knowledge of the existing research by focusing only on the latest, closely related, or reliable data. Only a comprehensive review article will go through the previous and available literature as much as possible and provide prospects.

Furthermore, Abrantes et al. criticized the appearance of old strains from the evolution/recombination of new strains [16]. As we mentioned above, these recombinations are representative of events, meaning that the major or minor parent may itself be the actual recombinant and only a representative of all the possible close relatives of an actual virus involved. In addition, a virus may exist undetected and identified later than the already published ones, and certain strains in a group may be included or excluded while updating the phylogenies and classification. Failure to understand these facts led the authors to misunderstand the overall evolution, clustering, recombination, and phylogeographic dynamics of the lagovirus presented in our analyses.

## Figures and Tables

**Figure 1:**
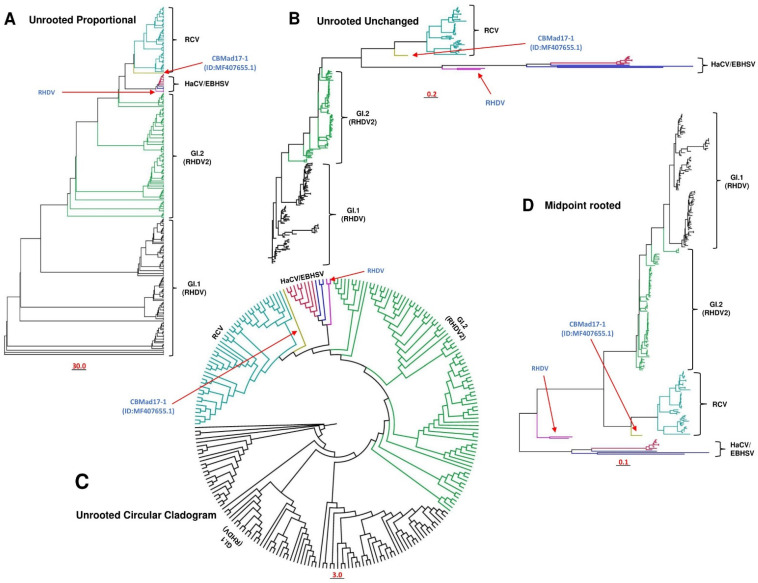
The full-length genome-based maximum likelihood phylogenetic tree of lagovirus. The unrooted tree set to proportional (**A**), the unrooted unchanged tree (**B**), the unrooted circular cladogram (**C**), and the midpoint-rooted tree (**D**) of all the lagoviruses (n = 233), excluding the FRG-USA strain (GenBank ID NC_001543.1), while including the FRG-Germany strain (GenBank ID M67473.1). The tree was inferred using the IQ tree v1.6.12 with 1000 bootstraps and the best-fitting model SYM+I+G4. In addition, the tree branches were also tested with the Shimodaira–Hasegawa-like approximate likelihood ratio test (SH-like aLRT) with 1000 replicates. The tree was visualized and modified using FigTree v1.4. Each clade is represented with a different color.

## Data Availability

The nucleotide sequence data used in this study are available in NCBI GenBank.

## Supplementary Materials

The following supporting information can be downloaded at: https://www.mdpi.com/article/10.3390/v16060928/s1, Figure S1: The full-length genomes-based maximum likelihood unrooted phylogenetic tree of lagovirus, which set to proportional.
