# Supplementary figures and images for: Reply to Abrantes et al. Recombination-Based Perspectives on Lagovirus Classification, Phylogenetic Patterns, and Evolutionary Dynamics. Comment on “Shah et al. Genetic Characteristics and Phylogeographic Dynamics of Lagoviruses, 1988–2021. Viruses 2023, 15, 815”
Source: Viruses. 2024 Jun 7;16(6):928. doi: 10.3390/v16060928 (PMC11209430; doi:10.3390/v16060928)

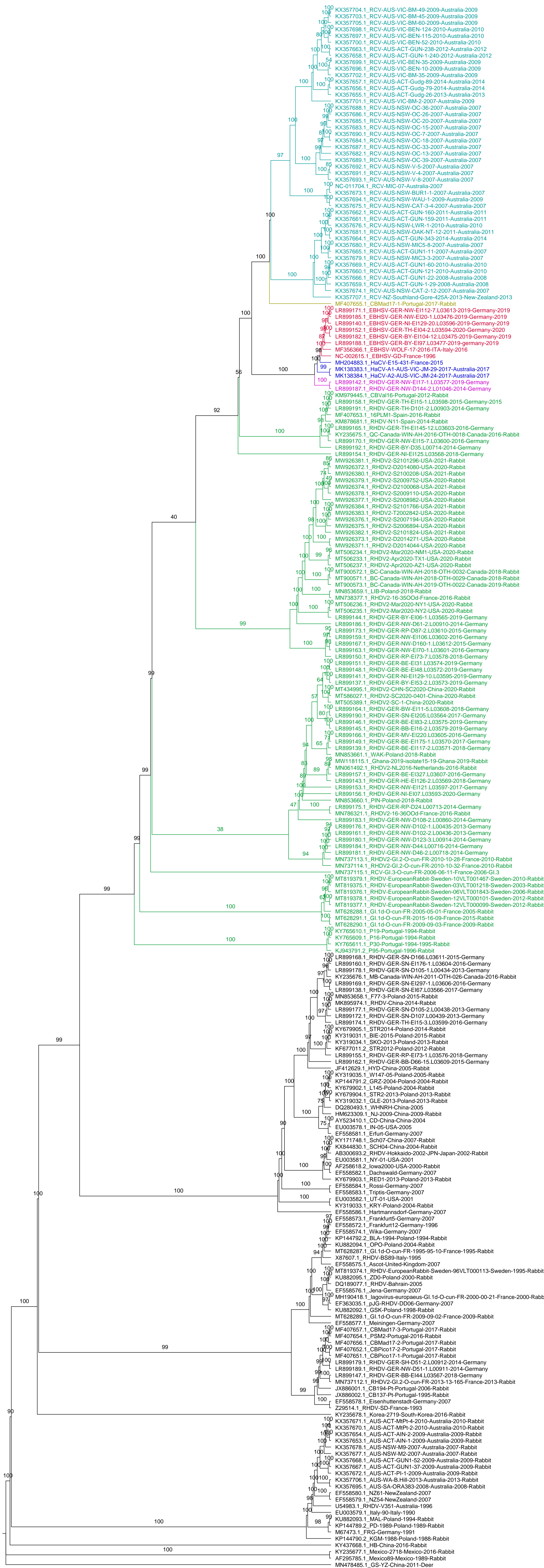

Supplement: Supplementary file 1 [file viruses-16-00928-s001.zip › viruses-3056985-supplementary.pdf]
